# Going with the Flo: The Role of Flo11-Dependent and Independent Interactions in Yeast Mat Formation

**DOI:** 10.3390/jof4040132

**Published:** 2018-12-07

**Authors:** Todd B. Reynolds

**Affiliations:** Department of Microbiology, University of Tennessee, Knoxville, TN 37996, USA; treynol6@utk.edu; Tel.: +1-865-974-4025

**Keywords:** Flo11, biofilm, mat formation, flocculins, adhesins

## Abstract

Strains of the bakers’ yeast *Saccharomyces cerevisiae* that are able to generate a multicellular structure called a mat on low percentage (0.3%) agar plates are given a selective advantage over strains that cannot exhibit this phenotype. This environment may exhibit some similarities to the rotting fruit on which *S. cerevisiae* often grows in nature. Mat formation occurs when the cells spread over the plate as they grow, and cells in the center of the biofilm aggregate to form multicellular structures that resemble a floral pattern. This multicellular behavior is dependent on the cell surface flocculin Flo11. This review covers recent information on the structure of Flo11 and how this likely impacts mat formation as well as how variegated expression of Flo11 influences mat formation. Finally, it also discusses several Flo11-independent genetic factors that control mat formation, such as vacuolar protein sorting (VPS) genes, cell wall signaling components, and heat shock proteins.

## 1. Introduction

The yeast *Saccharomyces cerevisiae* is able to generate a number of multicellular forms including flocs, invasive colonies, pseudohypae, biofilms, and mats. These multicellular forms give yeast selective advantages in specific growth conditions. All of these types of growth require the expression of cell wall protein adhesins referred to as flocculins that are generally not expressed in common laboratory strains like S288c [1,2,3]. However, many natural or industrial strains express these flocculin proteins allowing them to generate multicellular structures that give them selective advantages including promoting resistance to environmental stress and better nutrient acquisition.

**Figure 1:**
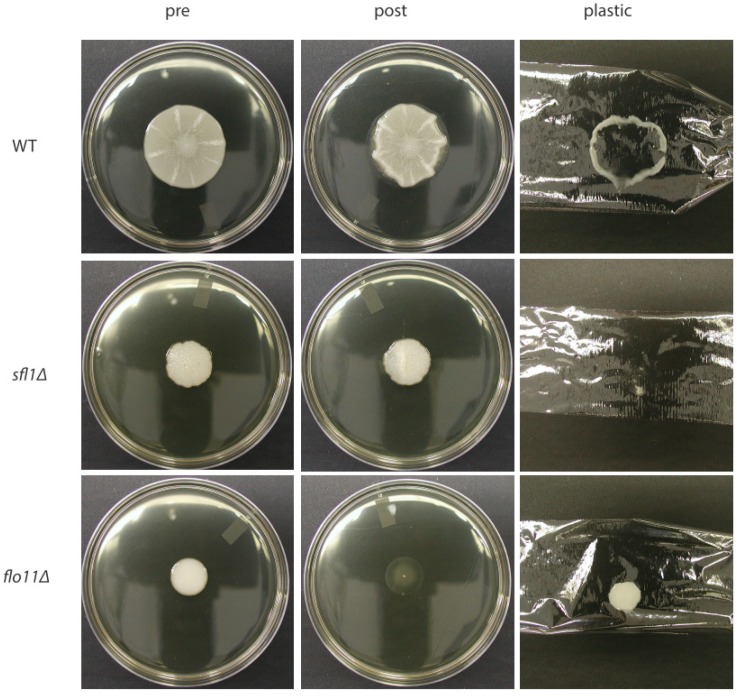
The *sfl1∆* mutant stays attached to the agar in the overlay adhesion assay and does not form a rim and hub like the wild-type, whereas the *flo11∆-* mutant does not adhere at all. Cells were grown for several days at ~25 °C on plates made with YPD and 0.3% agar. Then, plastic wrap is placed over the mat and removed. Cell growth prior to the assay is shown in the “pre” panels. Cells left on the agar following the assay are shown in the “post” panels. Cells that were removed with the plastic wrap are shown in the “plastic” column. WT = wild-type.

**Figure 2:**
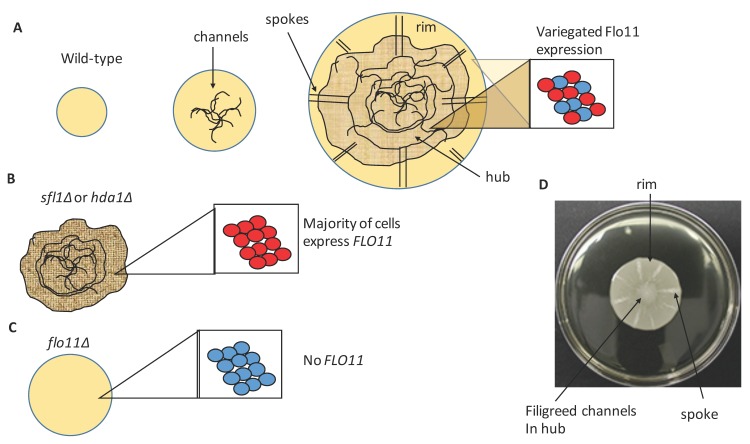
Mat formation is accompanied by the formation of channels that are correlated with variegated expression of Flo11. (**A**) A cartoon describes the formation of channels that correlate with cell adhesion during mat formation in wild-type and how some of these channels reach the edge of the mat and are referred to as spokes. The smooth edge of the mat lacks channels is referred to as the rim. (inset: cells from both the rim and hub appear to have variegated expression of Flo11 where red cells express Flo11 and blue do not express Flo11). (**B**) The sfl1∆ and hda1∆ mutants both exhibit expression of Flo11 by the majority of cells (inset: all red cells), and this correlates with them acting like hub cells with no rim and not spreading well. (**C**) A flo11∆ mutant looks more like a large rim with no hub and all cells lack Flo11 (inset: all blue cells). (**D**) A photo of a wild-type mat is shown and the different structures are shown to put the wild-type cartoon in (**A**) in better context.

Of these forms of growth, mat formation possibly resembles the most differentiated, and has been shown to give a yeast a significant growth advantage in specific environmental conditions compared to strains that are unable to generate mats [4,5]. Mats are formed when certain yeast strains grow on semi-solid, low percentage (0.3%) agar media (Figure 1 and Figure 2D); conditions that may have some resemblance to the rotting fruit on which these yeasts normally grow [6]. Mat formation was discovered in the yeast strain background ∑1278b [5], however this phenotype was observed in six out of 49 wild yeast isolates in one study [4], six out of 30 in another [7], and two out of five wine strains in a third study [8]. Thus, this is a phenotype that is common in natural yeast populations. Mats also may have some resemblance to the flor or velum biofilms formed by some yeast strains on the surface of sherry wine during its production [8,9,10,11]. 

Mat formation allows yeast cells to expand over large areas as they divide giving access to nutrients over a wide area [4,12]. Mats form over time on low percentage (0.3%) agar medium and are characterized by the formation of a floral-like biofilm that expands over the wet agar surface by sliding motility. As the mat grows, the center of the mat differentiates and generates a series of channels that form a filigreed appearance in the middle of the mat (the hub) while the edge (rim) continues to grow and is smooth in appearance (Figure 2A). As the mat enlarges past this point, in addition to the central filigreed channels, there are relatively straight channels that appear near the edge of the growing mat, and these are referred to as spokes (Figure 2A,D). The formation of the mat is not associated with differentiation of cells within different parts based on cellular morphology (e.g., yeast vs. pseudohyphae) [5]. However, there is a clear difference between the adhesion/aggregation behaviors of cells in different parts of the mat. Cells in the outer edge of the mat (rim) do not adhere to the agar, whereas cells in the filigreed inner section (hub) do adhere to the agar (WT in Figure 1) based on the overlay adhesion assay [12]. In addition, there is some reason to believe that the extracellular matrix may be deposited in the mat based on the appearance of an excreted form of the glycoprotein Flo11 found in the mat, which will be discussed further below [13]. 

## 2. Mat Formation Is Dependent on Flo11

**Figure 3:**
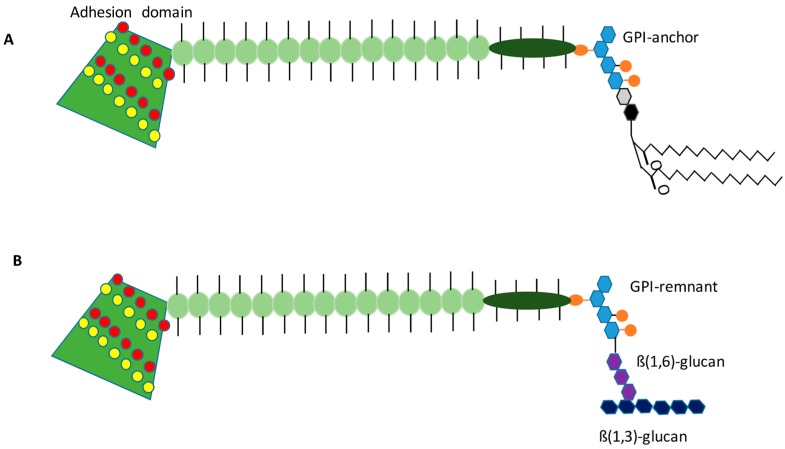
Flo11 appears to be a GPI-anchored protein with bands of hydrophobic residues in the N-terminal adhesion domain. The predicted Flo11 structure is shown in cartoon form, but lacking the N-terminal signal sequence, as it may be cleaved in the mature protein during translocation into the endoplasmic reticulum (ER). The N-terminal adhesion domain is wedge shaped and has two bands of hydrophobic, aromatic residues (yellow dots) as well as two bands of acidic residues (red dots). The serine-threonine-rich repeat regions in the stock are shown as light green balls with sticks representing predicted glycosylation. The dark green area proceeds the C-terminal GPI-anchor signal. (**A**) The membrane bound form of the protein with the full GPI-anchor is depicted. (**B**) The cell wall form of Flo11, bound by the GPI-remnant to the cell wall carbohydrates is depicted. Hexagons: The hexagons represent sugars in the GPI-anchor or cell wall. Light blue—mannose, Grey—*N*-glucosamine, Black—inositol, Purple—β(1,6)-glucan, Deep blue—β(1,3)-glucan. The orange circles on the GPI-anchor represent phosphoethanolamine groups.

Mat formation is absolutely dependent on the expression of the flocculin Flo11, as deletion of *FLO11* completely disrupts mat formation [5]. Flo11 is one of five flocculin genes (Flo1, Flo5, Flo9, Flo10, and Flo11) in *S. cerevisiae* that are involved in cell–cell/cell–surface adhesion [1,14]. These proteins are similar to adhesion proteins found in many other yeasts, including pathogens such as *Candida albicans* and *Candida glabrata*, and have a common structure [15,16]. These proteins consist of an N-terminal signal sequence, for entry into the secretory pathway, followed by an adhesion domain, then a series of repeated domains that are rich in serines and threonines that are heavily *O*-glycosylated, and finally a C-terminal glycosyphosphatidylinositol (GPI)-anchor signal sequence that mediates covalent attachment of the protein to a GPI-anchor in the endoplasmic reticulum (ER) [1,17] (Figure 3). Based on the model of other GPI-anchored proteins in yeast, Flo11 is delivered to the plasma membrane via membrane trafficking while attached to its GPI-anchor (Figure 3A). Then the GPI-anchor is hydrolyzed, and a remnant of the carbohydrate linkage is covalently attached to the cell wall, likely to β-1,6-glucan (Figure 3B) [18,19,20]. This GPI-protein is then able to interact outside the cell wall. This trafficking pattern has not been fully confirmed experimentally for Flo11. However, the protein can be extracted from the plasma membrane by detergent, and from the cell wall with β(1,3)-glucanase treatment [21]. These data thus suggest that the above model from other GPI-anchored cell wall proteins holds true for Flo11 as well.

A number of yeast flocculins, such as Flo1, Flo5, and Flo9, are lectins that bind to mannose carbohydrates in the cell wall to mediate flocculation. They are also sub-telomerically localized [1]. Flo11 differs from the others in that it is localized further from the telomere (~46 kb) on chromosome IX. Flo11 is predicted to be highly hydrophobic, and mediates homotypic binding and binding to hydrophobic surfaces like plastics [5,22,23]. 

A crystal structure of the N-terminal binding domain of Flo11 helped explain its hydrophobic binding characteristics [22]. The binding domain of Flo11 (Flo11A) forms a wedge shaped structure made up of anti-parallel beta sheets surrounded by alpha helices. The binding domain is enriched with exposed aromatic residues (tyrosines and tryptophans) in two bands on the surface that give it a strong hydrophobic quality (yellow dots on adhesion domain in Figure 3). This structure bears some resemblance to fungal hydrophobin proteins that are involved in sporulation of *Aspergillus* and other ascomycetes [22]. Despite the predominance of surface-exposed hydrophobic residues, Flo11 is very water-soluble. The hydrophobicity of the Flo11 binding domain helps explain why the presence of Flo11 strongly increases cell-surface hydrophobicity [5].

The hydrophobic interactions appear to mediate homotypic binding between Flo11 molecules on different cells, allowing aggregation, as well as adhesion of cells to agar. Mutations of hydrophobic residues to acidic residues greatly reduced Flo11–Flo11 interactions, and adhesion to agar [22]. Cells carrying Flo11 appear to be bound together by a thick fibrillar layer that is missing when Flo11 is disrupted, indicating that Flo11 forms a homotypic binding layer on the surface to mediate cell–cell interactions. This binding is not dependent on calcium, which is different from aggregation mediated by Flo1 and Flo10 [24]. 

## 3. Environmental Conditions Control Mat Formation

Flo11 is required for mat formation, and the homotypic interactions between Flo11 molecules on different cells likely mediate the ability of cells to aggregate and generate the channels that mark the adhesive hub of the biofilm [5]. Interestingly, the outside rim of the growing biofilm does not generate channels, and in fact, the cells do not adhere to the surface of the agar [12]. This can be measured by the overlay adhesion assay, where a piece of plastic wrap is placed over the top of the mat and removed, and then the smooth outer rim cells are selectively removed (first described in reference [12] and repeated in Figure 1). Glucose levels were measured at different points in the biofilm, and this revealed that glucose is consumed in a gradient as the mat grows in size, where levels are highest near the rim and lowest in the middle of the hub [12]. In addition, increased glucose levels in plates slow formation of the hub [12]. Furthermore, glucose starvation correlates with increased *FLO11* expression [12,25]. However, *FLO11* is highly expressed in both rim and hub cells [12], as cells in both populations are transcriptionally more like stationary phase cells than log phase cells [26]. In fact, microscopic examination of Flo11 protein expression via immunofluorescence reveals similar protein distribution in both rim and hub cells, despite the rim cells being non-adherent and the hub adhering tightly [12] (Figure 1 and Figure 2A). 

Another factor that can influence Flo11 function is pH of the media. A slight gradient develops in the mat as it grows, where the outside rim is at pH 5.0 and the very center of the hub is 4.7. Forcing the pH up to 5.8 (media pH) by buffering it with citrate buffer leads to less adhesion, although not a total loss, and lack of the channels characteristic of the rim. In contrast, buffering the pH to 4.9 with citrate buffer leads to formation of channels and loss of the non-adherent rim population [12]. This pH dependence may be due to the observation that the band of hydrophobic residues in the Flo11A domain are closely aligned with a band of acidic residues (red dots in Figure 3), and if the pH is raised above the pI of the protein to around pH 6.2, Flo11-dependent homotypic adhesion is lost, possibly due to electrostatic repulsion [22]. 

## 4. Cellular Flo11 Distribution Influences Mat Formation and Fitness in Low Agar Medium

The pH gradient within the mat is very slight, and based on the current data cannot fully explain the loss of adhesion that the rim experiences despite expressing Flo11 similarly to the hub. Another interesting observation is that Flo11 expression is variegated due to gene silencing, such that it is only expressed in approximately 40% of the population of cells in the rim or hub of mats [12] (Figure 2A). Interestingly, this variegation is stochastic, and if a single cell that is not expressing *FLO11* (*FLO11* silent) is chosen for analysis, within 1.8 cell divisions daughters will bud off that express *FLO11* [22,23]. Thus, individuals within the population can switch rapidly between expressing and being silenced. The loss of expression in many of the cells is due to gene silencing mediated by the histone deactylase protein Hda1 that is localized to the Flo11 promoter region by the Sfl1 transcription factor. Deletion of *SFL1* or *HDA1* leads to constitutive expression of Flo11 throughout the population resulting in much more adherent, flocculent cells [23] (Figure 1 and Figure 2B). 

It is surprising then, that the *sfl1∆* mutant, which overexpresses Flo11, behaves like a *flo11-* (*flo8∆*) mutant and has reduced fitness in low agar medium compared to wild-type [4]. Both *sfl1∆* and *flo8∆* mutants exhibit reduced ability to grow and spread (fitness), and a four-fold loss in biomass, specifically in this low agar environment. Both behave similarly to wild-type for growth and fitness on 2% agar medium and in liquid culture [4]. Thus, this growth disadvantage of the mutants is found in the low agar environment. Interestingly, *flo11∆* (behaves similarly to *flo8∆*) and *sfl1∆* strains differ greatly from one another and wild-type in their adhesion, as tested by the overlay adhesion assay. In the wild-type strain, the hub adheres to the agar, and rim is removed during the overlay adhesion assay (Figure 1) [12]. In contrast, the *flo11∆* strain is completely removed, but the *sfl1∆* mutant is the polar opposite, and sticks completely to the agar with no rim. The *flo11∆* strain also exhibits no patterns of aggregation, whereas *sfl1∆* resembles the hub (Figure 1 and Figure 2).

Thus, the population of cells with variegated expression of Flo11 appears to give yeast greater fitness in the environment of low agar plates. Regenberg et al. [4] tested to see if they could recreate the wild-type phenotype by mixing the *flo8∆* (*flo11-*) and *sfl1∆* cells together. They found that this led only to separate populations with aggregated cells in the middle and smooth cells around them, but together, they were unable to spread more than either mutant alone. Thus, the ability to generate a stochastic mixed population appears to be crucial for mat formation and fitness in this wet environment (Figure 2). 

In addition to Flo11 being expressed on the cell walls of yeast in a variegated manner, Flo11 is also found in a secreted form in biofilms that can be extracted by simply washing the cells [13,27]. Thus, Flo11 makes up part of the extracellular matrix of *Saccharomyces* biofilms. Its processing to create excreted Flo11 appears to depend at least in part on the Golgi-localized Kex2 protease, as well as the Rbd2 and Ecm14 proteases and the Spo1 phospholipase [13]. However, the function of secreted Flo11 in mat formation and other adhesion phenotypes is not understood. For example, does secreted Flo11 bind homotypically to cell-associated or even cell-wall bound Flo11? Does secreted Flo11 act as a surfactant? Is the secreted form important for biofilm spreading in the rim or adhesion to agar in the hub? What is the full mechanism for Flo11 release from the cell and excretion into the extracellular space?

## 5. Flo11 Expression Impacts Mat Formationf

The full literature that has explored the regulation of *FLO11* expression in yeast is too extensive to review here and is well-covered in other reviews [28,29,30,31]. However, much of the work in the area of *FLO11* expression has focused on pseudohyphal and invasive growth regulation. It is important to point out that a large-scale screen for mutations that impact invasive growth, pseudohyphal growth, and mat formation showed that mutations that disrupt one of these three phenotypes do not necessarily disrupt the other two [32]. For instance, the Protein kinase A (PKA) pathway, that acts downstream of yeast Ras2, is required for filamentous growth and invasive growth in *S. cerevisiae* [29,33,34,35]. The *tpk2∆* mutant, that blocks PKA signaling, blocks filamentous growth. However, while Ras2 is required for mat formation, *tpk2∆* does not impact this phenotype. The whole genome screen by Ryan et al. [32] revealed that there were at least 61 genes that impacted mat formation, filamentous growth, and invasive growth, and most of these affect *FLO11* expression. These include *FLO11*, *RPD3*, *SIN3*, *RXT2*, *RIM101*, *FLO8*, *TEC1*, *MSS11*, *MIT1*, and *MFG1*. However, there were also 306 mutations that perturbed mat formation but did not impact invasive growth or filamentous growth. Thus, there are genes not shared between these phenotypes. This is likely what explains the diversity of phenotypes that can be observed in natural isolates of *S. cerevisiae*, where some are able to undergo invasive growth or pseudohyphal growth, but cannot necessarily undergo mat formation [4,7,8]. Finally, another genetic factor that affects mat formation in ∑1278b is ploidy. As ploidy levels go up from haploid to diploid, triploid, and tetraploid, mat formation diminishes. This decrease in mat formation appears to be due to an inversely correlated decrease in *FLO11* expression that accompanies the increase in ploidy [5,36]. Thus, in natural strains, it is possible that the normally diploid strains will have decreased mat formation compared to haploid derivatives, but this remains to be confirmed.

## 6. Flo11 Is Necessary but Not Sufficient for Mat Formation

**Figure 4:**
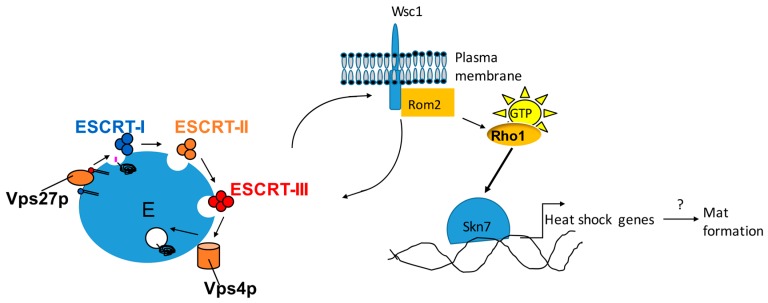
The endosomal trafficking machinery may influence mat formation by regulating Wsc1-dependent activation of Skn7 and downstream heat shock protein genes. This a speculative model based on current data. The endosomal trafficking machinery, of which 12 proteins are depicted, includes the ESCRT complexes which affect the trafficking of the Wsc1 protein which uses endosomal recycling to localize to the plasma membrane. Wsc1 is normally able to activate the Rom2 Guanylate Exchange Factor (GEF), which activates Rho1 by loading it with GTP (active-state). Active Rho1 is believed to affect activation of Skn7, a transcription factor that can express heat shock protein genes. Some heat shock proteins have been found to affect mat formation in a Flo11-independent manner.

While Flo11 is clearly necessary for mat formation, mutant studies have revealed that Flo11 is not sufficient for this phenotype [21,27,37,38]. A number of mutations have been generated that block mat formation, but do not affect Flo11 expression or invasive growth, including mutations in protein chaperones and the vacuolar protein sorting (vps) mutants that impact endosomal trafficking [39,40]. Specifically, a subset of vps mutants known as class E mutants block mat formation [27]. These class E mutants comprise the endosomal complexes required for transport (ESCRT) pathway, which is necessary for endosomal protein sorting. The ESCRT complex proteins consist of four complexes of proteins (I–IV) that work in succession to sort proteins into the multivesicular body (MVB) (Figure 4). The class E mutants can be further subdivided into class E1 and class E2 mutants based on their effects on mat formation and invasive growth [27]. Class E1 mutants (enriched in ESCRT complexes I, II, and part of III) block activation of the Rim101 transcription factor that is necessary for Flo11 expression [41], and therefore block other Flo11-dependent phenotypes like invasive growth [27]. Class E2 mutants (includes Vps27 and part of ESCRT III, as well as additional vps mutants) do not prohibit Flo11 expression or invasive growth, but do block mat formation [27]. The mechanism by which Class E2 mutants block mat formation is not yet clear, but may be through blocking the localization of the Wsc1 cell wall receptor, which shares this phenotype [21] (Figure 4). Wsc1 is at the head of a cell wall signaling pathway that includes the Slt2 MAPK pathway and may impact a pathway involving the Skn7 transcription factor [42,43]. Wsc1 activates the MAPK pathway by stimulating the Rom2 GDP exchange factor (GEF) to activate the Rho1 small GTPase which in turn activates both pathways downstream (Figure 4) [44,45]. Wsc1 is normally localized to specific points like the bud tip and bud neck on the plasma membrane by recycling action of the endosome [46]. Mutations in class E mutants disrupt this localization to the plasma membrane and trap Wsc1-GFP in the endosome. Disruption of *WSC1* blocks mat formation, but not invasive growth or Flo11 expression, while mutations in the Slt2 MAPK pathway components do not impact any of these phenotypes [21]. However, mutation of specific amino acid residues in Wsc1 that interact with Rom2 [44,45] disrupt mat formation but not invasive growth or Flo11 [21]. This suggests that Wsc1 is required for mat formation, but is not acting through the downstream Slt2 kinase. Alternatively, it could be acting through the Skn7 transcription factor, as disruption of *SKN7* leads to a Class E2 like phenotype, suggesting that this is the case [21]. 

The mechanism by which Skn7 regulates mat formation is unknown, however one potential target is related to heat shock proteins. A target gene of the Skn7 transcription factor during oxidative stress is the Ssa1 Hsp70 heat shock protein gene [47]. Furthermore, Skn7 has been shown to regulate a number of heat shock genes in response to oxidative stress, including *SSA1*, *HSP12*, and *HSP26*, although there are discrepancies for *SSA1* between two studies [47,48]. Several heat shock genes, including *HSP12*, *HSP26*, and two Hsp70 homologs of *SSA1* called *SSA3* and *SSE2*, were all upregulated during mat formation [26]. Moreover, disruption of *SSA1* and several related Hsp70 genes in *S. cerevisiae*, including *SSA3*, diminish mat formation, but not Flo11 expression or invasive growth [37,38]. Thus, it is conceivable that Ssa1 and other heat shock proteins are regulated by Skn7 in response to mat formation conditions, and this could help explain the defects seen in the Wsc1 and vps mutants (Figure 4). However, direct links between these heat shock genes and signaling pathways in this model have yet to be shown, and this may not be the explanation. Many questions remain. Is the defect in mat formation caused by vps mutants directly related to Wsc1 function or are these separate parallel effects? Is Skn7 acting downstream of Wsc1 to mediate mat formation? What is the role of Ssa1 and other heat shock proteins in mat formation? In *Candida albicans*, Ssa homologs have been shown to be localized in the cell wall and are thought to be delivered by a non-canonical secretion mechanism such as exosomes [49,50,51]. The ESCRT complexes have a role in exosome formation, and therefore could potentially be involved in delivering proteins such as Ssa1 to the cell wall or extracellular space, but this is all highly speculative. 

## 7. Conclusions

Mat formation represents a form of biofilm formation in yeast that gives these microbes a growth advantage in wet, semisolid conditions, which may be similar to what they experience in rotting fruit where they tend to thrive [4,5,12]. Mats represent a complex form of multicellular growth and much remains to be learned about how the adhesin Flo11 facilitates this process. Large questions revolve around how Flo11 interacts at the cell surface, the function and processing of secreted Flo11, and the reason that epigenetic silencing of Flo11 is necessary for mat formation. In addition, there are many unanswered questions regarding genes and proteins that are necessary for mat formation apart from Flo11 such as the VPS, Wsc1, Skn7, and chaperones. Answering these questions will assist in understanding this interesting form of multicellular growth.
